# Crystal structure of 3-(4-methyl­phen­yl)-1-phenyl-5-[(*E*)-2-phenyl­ethen­yl]-1*H*-pyrazole

**DOI:** 10.1107/S2056989015022811

**Published:** 2015-12-06

**Authors:** Farook Adam, Sharath Poojary Charishma, Basrur Ramya Prabhu, Seranthimata Samshuddin, Nadiah Ameram

**Affiliations:** a School of Chemical Sciences,Universiti Sains Malaysia, 18000, Pulau Pinang, Malaysia; b Department of P.G. Studies in Chemistry, Alvas College, Moodbidri, Karnataka, 574 227, India

**Keywords:** crystal structure, pyrazole, π–π stacking inter­actions

## Abstract

In the title compound, C_24_H_20_N_2_, the dihedral angles between the pyrazole ring and the pendant phenyl, toluoyl and phenyl­ethenyl rings are 41.50 (8), 4.41 (8) and 31.07 (8)°, respectively. In the crystal, inversion dimers linked by a π–π stacking inter­actions between the phenyl­ethenyl rings are observed [centroid–centroid separation = 3.5857 (9) Å].

## Related literature   

For background to pyrazoles, see: Samshuddin *et al.* (2012 ▸); Sarojini *et al.* (2010 ▸); For related crystal structures, see: Jasinski *et al.* (2012 ▸); Baktır *et al.* (2011 ▸).
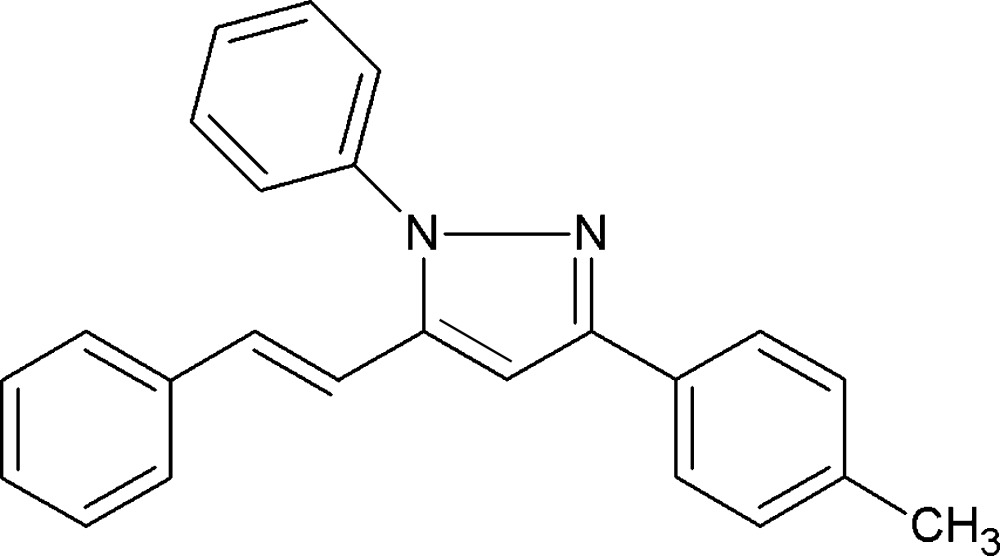


## Experimental   

### Crystal data  

C_24_H_20_N_2_
*M* *_r_* = 336.42
Monoclinic, 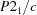

*a* = 9.6470 (8) Å
*b* = 14.1077 (12) Å
*c* = 14.0062 (12) Å
β = 104.891 (1)°
*V* = 1842.2 (3) Å^3^
*Z* = 4
Mo *K*α radiation
μ = 0.07 mm^−1^
*T* = 100 K
0.38 × 0.24 × 0.14 mm

### Data collection  

Bruker APEXII CCD diffractometer
Absorption correction: multi-scan (*SADABS*; Bruker, 2014 ▸) *T*_min_ = 0.915, *T*_max_ = 0.963
32138 measured reflections
5495 independent reflections
4226 reflections with *I* > 2σ(*I*)
*R*_int_ = 0.033

### Refinement  

*R*[*F* ^2^ > 2σ(*F* ^2^)] = 0.061
*wR*(*F* ^2^) = 0.167
*S* = 1.04
5495 reflections
236 parameters
H-atom parameters constrained
Δρ_max_ = 0.57 e Å^−3^
Δρ_min_ = −0.24 e Å^−3^

### 

Data collection: *APEX2* (Bruker, 2014 ▸); cell refinement: *SAINT* (Bruker, 2014 ▸); data reduction: *SAINT*; program(s) used to solve structure: *SHELXS97* (Sheldrick 2008 ▸); program(s) used to refine structure: *SHELXL2014* (Sheldrick, 2015 ▸); molecular graphics: *SHELXTL* (Sheldrick, 2008 ▸); software used to prepare material for publication: *SHELXTL*.

## Supplementary Material

Crystal structure: contains datablock(s) I, New_Global_Publ_Block. DOI: 10.1107/S2056989015022811/hb7552sup1.cif

Structure factors: contains datablock(s) I. DOI: 10.1107/S2056989015022811/hb7552Isup2.hkl

Supporting information file. DOI: 10.1107/S2056989015022811/hb7552Isup3.cml

**Fig. 1.**
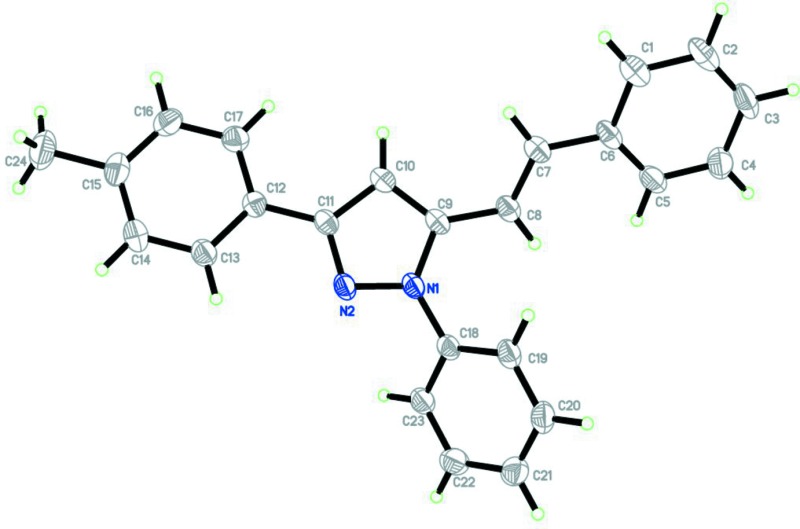
A view of the molecular structure of the title compound, showing the atom labelling. Displacement ellipsoids are drawn at the 50% probability level.

**Fig. 2.**
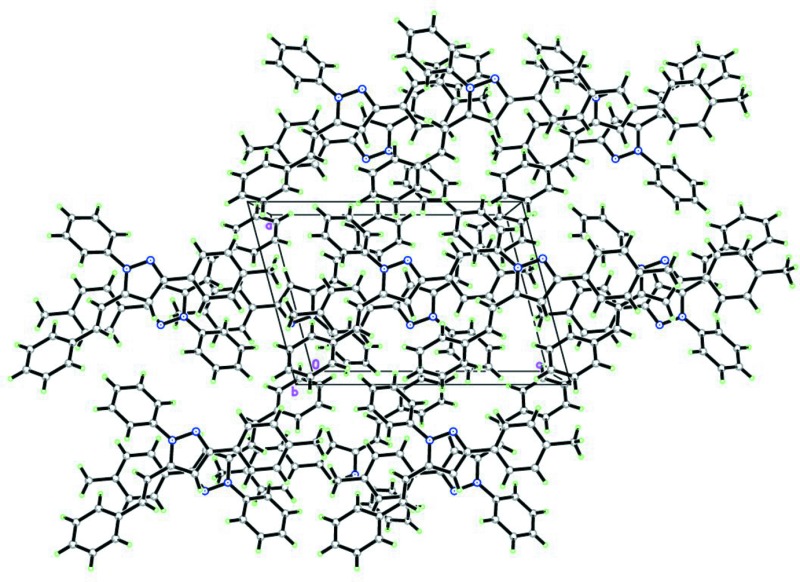
A view along the a axis of the crystal packing of the title compound.

. DOI: 10.1107/S2056989015022811/hb7552fig1.tif
A view of the molecular structure of the title compound, showing the atom labelling. Displacement ellipsoids are drawn at the 50% probability level.

a . DOI: 10.1107/S2056989015022811/hb7552fig2.tif
A view along the *a* axis of the crystal packing of the title compound.

CCDC reference: 1439397

Additional supporting information:  crystallographic information; 3D view; checkCIF report

## supplementary crystallographic information

### S1. Comment

Pyrazoles are well known as important structural units in a wide variety of biologically active natural products as well as useful synthetic intermediates (Sarojini *et al.*, 2010; Samshuddin *et al.*, 2012). Many 1,3,5-triaryl-2-pyrazolines were utilized in industries as scintillation solutes and as fluorescent agents. The crystal structures of some pyrazolines *viz*., 3,5-bis(4-methylphenyl)-1-phenyl-4,5-dihydro-1*H*-pyrazole, 3,5-bis(4-methoxyphenyl)-1-phenyl-4,5-dihydro-1*H*-pyrazole (Baktir *et al.*, 2011) have been reported. In view of the importance of pyrazolines, the title compound (I) is prepared and its crystal structure is reported.

### S2. Experimental

A mixture of (2*E*,4*E*)-1-(4-methylphenyl)-5-phenylpenta-2,4-dien-1-one (2.48 g, 0.01 mol) and phenylhydrazine (1.08 g,0.01 mol) in 30 ml acetic acid was refluxed for 10 h. The reaction mixture was cooled and poured into 500 ml ice-cold water. The precipitate was collected by filtration and purified by recrystallization from ethanol. Colourless blocks were grown from acetone solution by slow evaporation; m. p. 471–474 K. Yield: 63%.

### S3. Refinement

H atoms were placed in calculated positions and refined as riding with C–H = 0.95 Å (0.98 Å for methyl H atoms) and *U*_iso_(H) = 1.2*U*_eq_(C) or 1.5*U*_eq_(C_methyl_).

### Crystal data

**Table:**

C_24_H_20_N_2_	*D*_x_ = 1.213 Mg m^−^^3^
*M**_r_* = 336.42	Melting point = 471–474 K
Monoclinic, *P*2_1_/*c*	Mo *K*α radiation, λ = 0.71073 Å
*a* = 9.6470 (8) Å	Cell parameters from 9097 reflections
*b* = 14.1077 (12) Å	θ = 2.2–30.1°
*c* = 14.0062 (12) Å	µ = 0.07 mm^−^^1^
β = 104.891 (1)°	*T* = 100 K
*V* = 1842.2 (3) Å^3^	Block, colourless
*Z* = 4	0.38 × 0.24 × 0.14 mm
*F*(000) = 712	

### Data collection

**Table:**

Bruker APEXII CCD diffractometer	4226 reflections with *I* > 2σ(*I*)
φ and ω scans	*R*_int_ = 0.033
Absorption correction: multi-scan (*SADABS*; Bruker, 2014)	θ_max_ = 30.3°, θ_min_ = 2.1°
*T*_min_ = 0.915, *T*_max_ = 0.963	*h* = −13→13
32138 measured reflections	*k* = −19→20
5495 independent reflections	*l* = −19→19

### Refinement

**Table:**

Refinement on *F*^2^	0 restraints
Least-squares matrix: full	Hydrogen site location: inferred from neighbouring sites
*R*[*F*^2^ > 2σ(*F*^2^)] = 0.061	H-atom parameters constrained
*wR*(*F*^2^) = 0.167	*w* = 1/[σ^2^(*F*_o_^2^) + (0.0767*P*)^2^ + 0.886*P*] where *P* = (*F*_o_^2^ + 2*F*_c_^2^)/3
*S* = 1.04	(Δ/σ)_max_ < 0.001
5495 reflections	Δρ_max_ = 0.57 e Å^−^^3^
236 parameters	Δρ_min_ = −0.24 e Å^−^^3^

### Special details

**Table:**

Geometry. All e.s.d.'s (except the e.s.d. in the dihedral angle between two l.s. planes) are estimated using the full covariance matrix. The cell e.s.d.'s are taken into account individually in the estimation of e.s.d.'s in distances, angles and torsion angles; correlations between e.s.d.'s in cell parameters are only used when they are defined by crystal symmetry. An approximate (isotropic) treatment of cell e.s.d.'s is used for estimating e.s.d.'s involving l.s. planes.

### Fractional atomic coordinates and isotropic or equivalent isotropic displacement parameters (Å^2^)

**Table:**

	*x*	*y*	*z*	*U*_iso_*/*U*_eq_	
N1	0.64167 (12)	0.60320 (9)	0.42861 (8)	0.0265 (3)	
N2	0.69140 (12)	0.59884 (9)	0.52903 (8)	0.0269 (3)	
C1	0.10080 (17)	0.75054 (12)	0.13886 (13)	0.0383 (4)	
H1A	0.0740	0.7816	0.1916	0.046*	
C2	0.02522 (18)	0.76888 (14)	0.04221 (14)	0.0437 (4)	
H2A	−0.0527	0.8122	0.0295	0.052*	
C3	0.06214 (17)	0.72491 (14)	−0.03526 (13)	0.0423 (4)	
H3A	0.0112	0.7387	−0.1013	0.051*	
C4	0.17484 (18)	0.65993 (15)	−0.01636 (13)	0.0417 (4)	
H4A	0.1995	0.6282	−0.0695	0.050*	
C5	0.25128 (16)	0.64156 (12)	0.08087 (12)	0.0355 (4)	
H5A	0.3284	0.5976	0.0934	0.043*	
C6	0.21586 (14)	0.68694 (11)	0.15970 (11)	0.0296 (3)	
C7	0.29464 (15)	0.67433 (11)	0.26385 (11)	0.0298 (3)	
H7A	0.2541	0.7019	0.3124	0.036*	
C8	0.41821 (15)	0.62750 (11)	0.29599 (11)	0.0275 (3)	
H8A	0.4572	0.5962	0.2488	0.033*	
C9	0.49623 (14)	0.62204 (10)	0.39980 (10)	0.0254 (3)	
C10	0.45197 (14)	0.63017 (10)	0.48572 (10)	0.0261 (3)	
H10A	0.3578	0.6433	0.4913	0.031*	
C11	0.57660 (14)	0.61475 (10)	0.56396 (10)	0.0242 (3)	
C12	0.59023 (15)	0.61272 (10)	0.67117 (10)	0.0249 (3)	
C13	0.72390 (16)	0.60008 (11)	0.73861 (11)	0.0302 (3)	
H13A	0.8072	0.5928	0.7150	0.036*	
C14	0.73624 (18)	0.59809 (12)	0.83953 (11)	0.0359 (4)	
H14A	0.8279	0.5893	0.8840	0.043*	
C15	0.61633 (19)	0.60878 (12)	0.87664 (12)	0.0370 (4)	
C16	0.48304 (19)	0.61886 (13)	0.80913 (12)	0.0377 (4)	
H16A	0.3994	0.6243	0.8327	0.045*	
C17	0.46994 (16)	0.62111 (11)	0.70806 (11)	0.0317 (3)	
H17A	0.3779	0.6284	0.6637	0.038*	
C18	0.74029 (14)	0.59214 (10)	0.37003 (10)	0.0261 (3)	
C19	0.73476 (15)	0.65173 (11)	0.28996 (10)	0.0296 (3)	
H19A	0.6638	0.6999	0.2731	0.036*	
C20	0.83463 (17)	0.63971 (12)	0.23509 (11)	0.0344 (3)	
H20A	0.8307	0.6791	0.1795	0.041*	
C21	0.94044 (17)	0.57045 (13)	0.26085 (12)	0.0361 (4)	
H21A	1.0084	0.5627	0.2230	0.043*	
C22	0.94626 (17)	0.51269 (12)	0.34226 (12)	0.0358 (3)	
H22A	1.0196	0.4662	0.3607	0.043*	
C23	0.84600 (15)	0.52252 (11)	0.39652 (11)	0.0302 (3)	
H23A	0.8490	0.4822	0.4514	0.036*	
C24	0.6314 (3)	0.61195 (17)	0.98668 (13)	0.0542 (5)	
H24A	0.7114	0.5714	1.0205	0.081*	
H24B	0.5426	0.5893	1.0006	0.081*	
H24C	0.6500	0.6773	1.0103	0.081*	

### Atomic displacement parameters (Å^2^)

**Table:**

	*U*^11^	*U*^22^	*U*^33^	*U*^12^	*U*^13^	*U*^23^
N1	0.0212 (5)	0.0337 (6)	0.0215 (5)	−0.0003 (4)	0.0000 (4)	0.0022 (4)
N2	0.0236 (5)	0.0334 (7)	0.0207 (5)	−0.0019 (4)	0.0000 (4)	0.0029 (4)
C1	0.0330 (8)	0.0356 (9)	0.0409 (9)	0.0033 (6)	0.0000 (6)	0.0059 (7)
C2	0.0317 (8)	0.0431 (10)	0.0480 (10)	0.0059 (7)	−0.0049 (7)	0.0160 (8)
C3	0.0286 (7)	0.0552 (11)	0.0353 (8)	−0.0063 (7)	−0.0061 (6)	0.0203 (8)
C4	0.0344 (8)	0.0574 (11)	0.0316 (8)	−0.0025 (7)	0.0052 (6)	0.0101 (7)
C5	0.0247 (6)	0.0409 (9)	0.0377 (8)	0.0027 (6)	0.0024 (6)	0.0123 (7)
C6	0.0213 (6)	0.0294 (7)	0.0325 (7)	−0.0036 (5)	−0.0033 (5)	0.0090 (6)
C7	0.0256 (6)	0.0299 (7)	0.0306 (7)	0.0013 (5)	0.0014 (5)	0.0007 (6)
C8	0.0231 (6)	0.0298 (7)	0.0265 (7)	−0.0018 (5)	0.0007 (5)	0.0027 (5)
C9	0.0221 (6)	0.0241 (7)	0.0262 (6)	0.0012 (5)	−0.0005 (5)	0.0006 (5)
C10	0.0234 (6)	0.0241 (7)	0.0278 (7)	0.0015 (5)	0.0010 (5)	−0.0001 (5)
C11	0.0230 (6)	0.0218 (6)	0.0255 (6)	−0.0011 (5)	0.0018 (5)	0.0005 (5)
C12	0.0266 (6)	0.0217 (6)	0.0248 (6)	−0.0010 (5)	0.0040 (5)	−0.0015 (5)
C13	0.0265 (6)	0.0347 (8)	0.0276 (7)	−0.0018 (5)	0.0035 (5)	0.0007 (6)
C14	0.0371 (8)	0.0388 (9)	0.0271 (7)	−0.0030 (6)	−0.0004 (6)	−0.0002 (6)
C15	0.0484 (9)	0.0354 (8)	0.0265 (7)	0.0006 (7)	0.0086 (6)	−0.0029 (6)
C16	0.0405 (8)	0.0409 (9)	0.0346 (8)	0.0071 (7)	0.0150 (7)	−0.0015 (7)
C17	0.0305 (7)	0.0319 (8)	0.0317 (7)	0.0041 (6)	0.0065 (6)	−0.0027 (6)
C18	0.0206 (6)	0.0323 (7)	0.0227 (6)	−0.0040 (5)	0.0004 (5)	0.0003 (5)
C19	0.0268 (6)	0.0341 (8)	0.0240 (6)	−0.0040 (5)	−0.0004 (5)	0.0028 (5)
C20	0.0355 (8)	0.0434 (9)	0.0221 (6)	−0.0076 (6)	0.0032 (5)	0.0023 (6)
C21	0.0331 (7)	0.0455 (9)	0.0300 (7)	−0.0048 (7)	0.0086 (6)	−0.0030 (7)
C22	0.0291 (7)	0.0394 (9)	0.0376 (8)	0.0009 (6)	0.0061 (6)	−0.0003 (7)
C23	0.0250 (6)	0.0352 (8)	0.0280 (7)	−0.0013 (6)	0.0024 (5)	0.0042 (6)
C24	0.0701 (13)	0.0646 (13)	0.0279 (8)	0.0045 (11)	0.0123 (8)	−0.0029 (8)

### Geometric parameters (Å, º)

**Table:**

N1—N2	1.3657 (15)	C12—C13	1.4003 (19)
N1—C9	1.3824 (17)	C13—C14	1.388 (2)
N1—C18	1.4156 (18)	C13—H13A	0.9500
N2—C11	1.3397 (18)	C14—C15	1.393 (2)
C1—C2	1.387 (2)	C14—H14A	0.9500
C1—C6	1.398 (2)	C15—C16	1.394 (2)
C1—H1A	0.9500	C15—C24	1.511 (2)
C2—C3	1.374 (3)	C16—C17	1.389 (2)
C2—H2A	0.9500	C16—H16A	0.9500
C3—C4	1.394 (3)	C17—H17A	0.9500
C3—H3A	0.9500	C18—C19	1.392 (2)
C4—C5	1.396 (2)	C18—C23	1.396 (2)
C4—H4A	0.9500	C19—C20	1.389 (2)
C5—C6	1.393 (2)	C19—H19A	0.9500
C5—H5A	0.9500	C20—C21	1.392 (2)
C6—C7	1.472 (2)	C20—H20A	0.9500
C7—C8	1.336 (2)	C21—C22	1.391 (2)
C7—H7A	0.9500	C21—H21A	0.9500
C8—C9	1.4574 (19)	C22—C23	1.382 (2)
C8—H8A	0.9500	C22—H22A	0.9500
C9—C10	1.381 (2)	C23—H23A	0.9500
C10—C11	1.4201 (18)	C24—H24A	0.9800
C10—H10A	0.9500	C24—H24B	0.9800
C11—C12	1.4736 (19)	C24—H24C	0.9800
C12—C17	1.391 (2)		
			
N2—N1—C9	111.77 (11)	C14—C13—C12	120.84 (14)
N2—N1—C18	118.78 (11)	C14—C13—H13A	119.6
C9—N1—C18	129.41 (12)	C12—C13—H13A	119.6
C11—N2—N1	105.30 (11)	C13—C14—C15	121.05 (15)
C2—C1—C6	120.89 (17)	C13—C14—H14A	119.5
C2—C1—H1A	119.6	C15—C14—H14A	119.5
C6—C1—H1A	119.6	C14—C15—C16	117.89 (14)
C3—C2—C1	120.57 (16)	C14—C15—C24	120.83 (16)
C3—C2—H2A	119.7	C16—C15—C24	121.27 (17)
C1—C2—H2A	119.7	C17—C16—C15	121.34 (15)
C2—C3—C4	119.65 (15)	C17—C16—H16A	119.3
C2—C3—H3A	120.2	C15—C16—H16A	119.3
C4—C3—H3A	120.2	C16—C17—C12	120.70 (14)
C3—C4—C5	119.85 (18)	C16—C17—H17A	119.7
C3—C4—H4A	120.1	C12—C17—H17A	119.7
C5—C4—H4A	120.1	C19—C18—C23	120.89 (14)
C6—C5—C4	120.81 (15)	C19—C18—N1	120.55 (13)
C6—C5—H5A	119.6	C23—C18—N1	118.52 (13)
C4—C5—H5A	119.6	C20—C19—C18	118.94 (14)
C5—C6—C1	118.21 (14)	C20—C19—H19A	120.5
C5—C6—C7	124.10 (13)	C18—C19—H19A	120.5
C1—C6—C7	117.67 (15)	C19—C20—C21	120.61 (14)
C8—C7—C6	125.62 (15)	C19—C20—H20A	119.7
C8—C7—H7A	117.2	C21—C20—H20A	119.7
C6—C7—H7A	117.2	C22—C21—C20	119.75 (15)
C7—C8—C9	123.35 (14)	C22—C21—H21A	120.1
C7—C8—H8A	118.3	C20—C21—H21A	120.1
C9—C8—H8A	118.3	C23—C22—C21	120.36 (15)
C10—C9—N1	106.23 (11)	C23—C22—H22A	119.8
C10—C9—C8	131.97 (13)	C21—C22—H22A	119.8
N1—C9—C8	121.78 (13)	C22—C23—C18	119.42 (14)
C9—C10—C11	105.64 (12)	C22—C23—H23A	120.3
C9—C10—H10A	127.2	C18—C23—H23A	120.3
C11—C10—H10A	127.2	C15—C24—H24A	109.5
N2—C11—C10	111.06 (12)	C15—C24—H24B	109.5
N2—C11—C12	120.22 (12)	H24A—C24—H24B	109.5
C10—C11—C12	128.72 (13)	C15—C24—H24C	109.5
C17—C12—C13	118.14 (13)	H24A—C24—H24C	109.5
C17—C12—C11	120.82 (13)	H24B—C24—H24C	109.5
C13—C12—C11	121.02 (13)		
			
C9—N1—N2—C11	0.18 (16)	C10—C11—C12—C17	4.1 (2)
C18—N1—N2—C11	177.93 (12)	N2—C11—C12—C13	4.0 (2)
C6—C1—C2—C3	−0.1 (3)	C10—C11—C12—C13	−177.46 (14)
C1—C2—C3—C4	1.2 (3)	C17—C12—C13—C14	−1.4 (2)
C2—C3—C4—C5	−1.4 (3)	C11—C12—C13—C14	−179.92 (14)
C3—C4—C5—C6	0.4 (3)	C12—C13—C14—C15	−0.1 (3)
C4—C5—C6—C1	0.7 (2)	C13—C14—C15—C16	1.8 (3)
C4—C5—C6—C7	−177.47 (15)	C13—C14—C15—C24	−176.62 (17)
C2—C1—C6—C5	−0.8 (2)	C14—C15—C16—C17	−1.9 (3)
C2—C1—C6—C7	177.42 (15)	C24—C15—C16—C17	176.47 (17)
C5—C6—C7—C8	8.2 (2)	C15—C16—C17—C12	0.4 (3)
C1—C6—C7—C8	−169.96 (16)	C13—C12—C17—C16	1.3 (2)
C6—C7—C8—C9	176.13 (14)	C11—C12—C17—C16	179.79 (14)
N2—N1—C9—C10	0.13 (16)	N2—N1—C18—C19	−136.00 (14)
C18—N1—C9—C10	−177.32 (14)	C9—N1—C18—C19	41.3 (2)
N2—N1—C9—C8	−178.33 (13)	N2—N1—C18—C23	41.74 (19)
C18—N1—C9—C8	4.2 (2)	C9—N1—C18—C23	−140.95 (15)
C7—C8—C9—C10	24.7 (3)	C23—C18—C19—C20	1.3 (2)
C7—C8—C9—N1	−157.24 (15)	N1—C18—C19—C20	179.02 (13)
N1—C9—C10—C11	−0.37 (15)	C18—C19—C20—C21	−1.3 (2)
C8—C9—C10—C11	177.87 (15)	C19—C20—C21—C22	0.0 (2)
N1—N2—C11—C10	−0.42 (16)	C20—C21—C22—C23	1.2 (2)
N1—N2—C11—C12	178.39 (12)	C21—C22—C23—C18	−1.2 (2)
C9—C10—C11—N2	0.50 (16)	C19—C18—C23—C22	−0.1 (2)
C9—C10—C11—C12	−178.17 (13)	N1—C18—C23—C22	−177.85 (13)
N2—C11—C12—C17	−174.51 (14)		
