# Supplementary figures and images for: Crystal structure of 3-(4-methyl­phen­yl)-1-phenyl-5-[(E)-2-phenyl­ethen­yl]-1H-pyrazole
Source: Acta Crystallogr E Crystallogr Commun. 2015 Dec 6;71(Pt 12):o1020. doi: 10.1107/S2056989015022811 (PMC4719956; doi:10.1107/S2056989015022811)

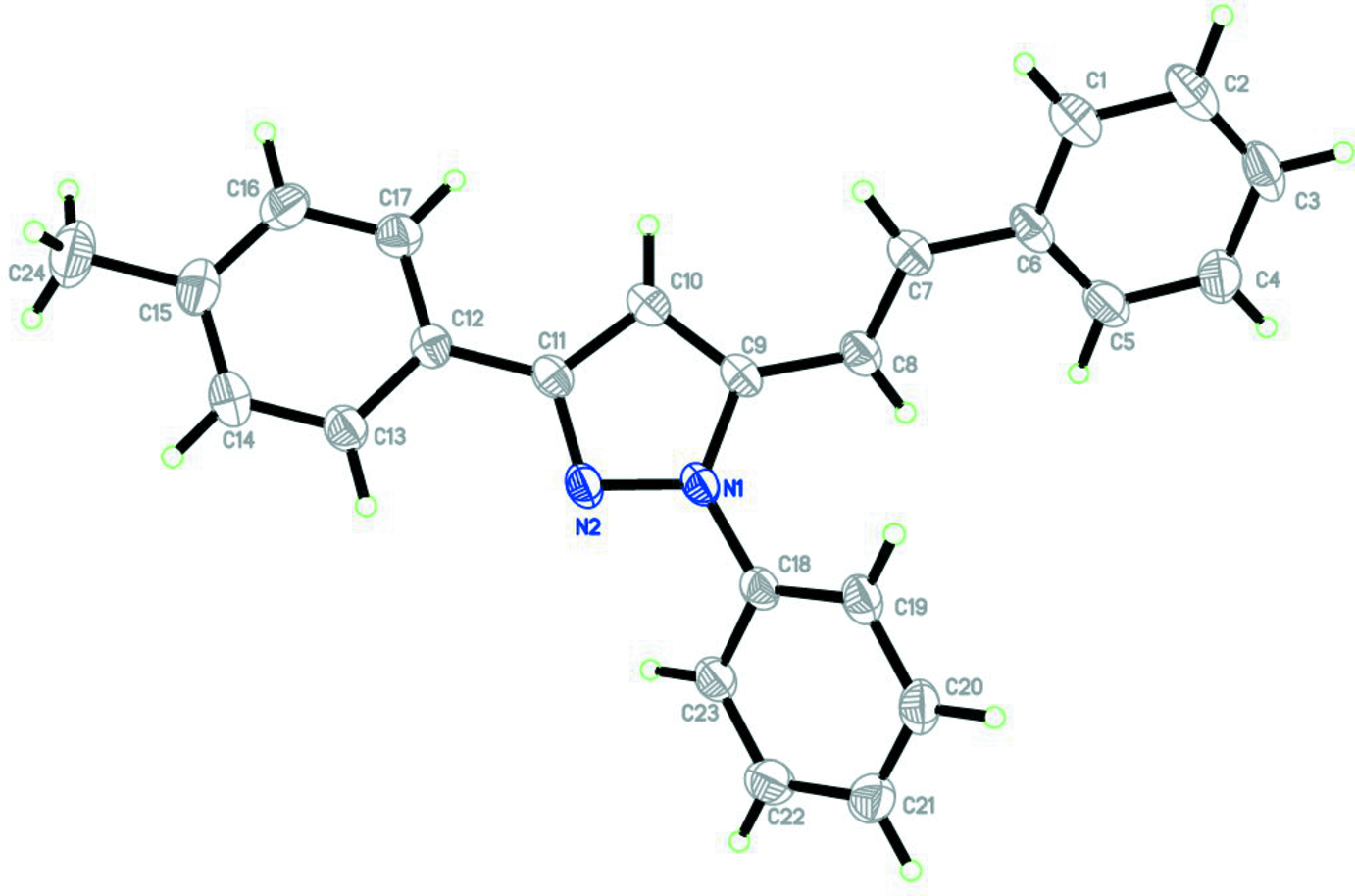

Supplement: Supplementary file 4 [file e-71-o1020-fig1.tif]

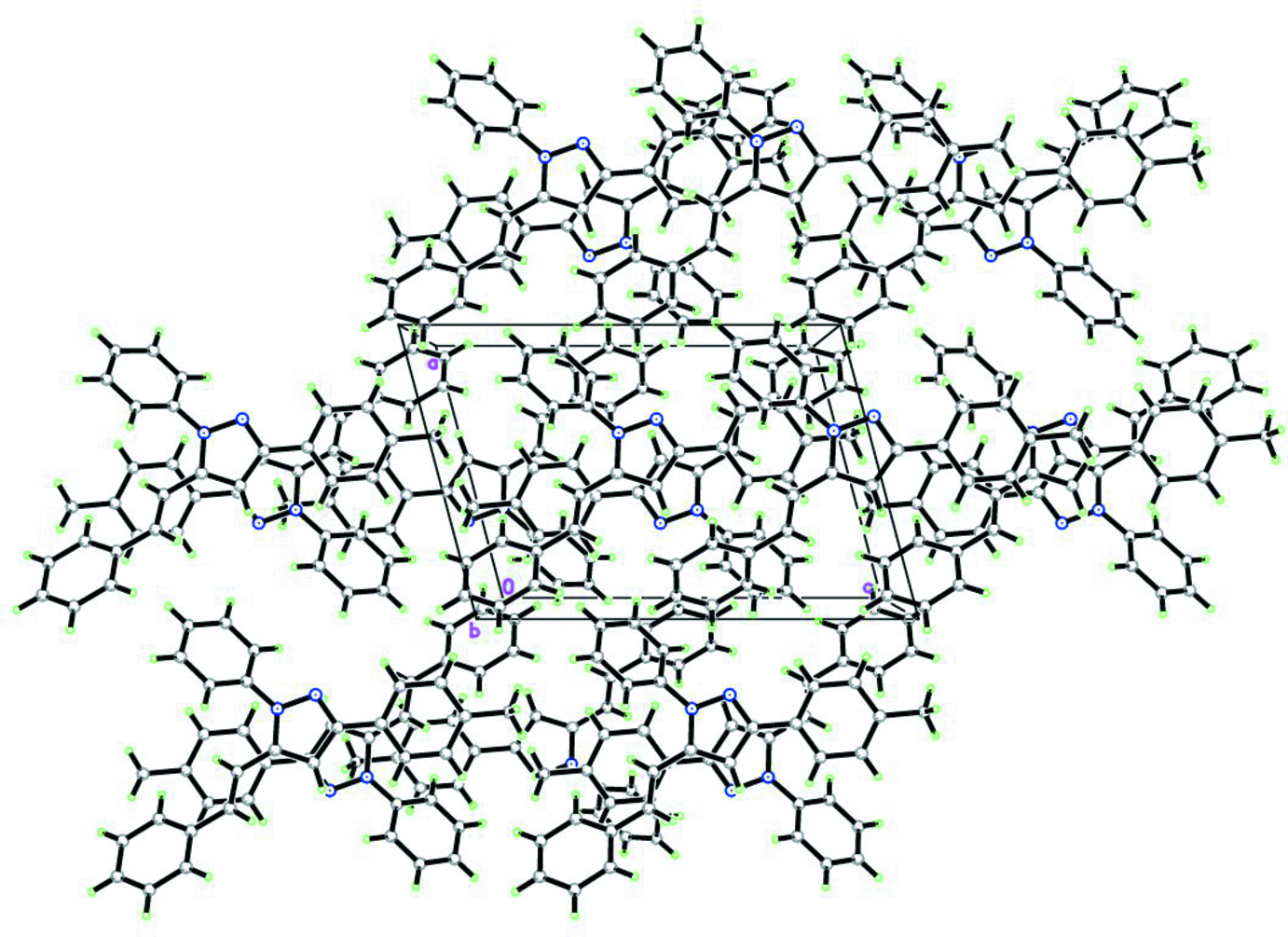

Supplement: Supplementary file 5 [file e-71-o1020-fig2.tif]
